# Perspectives of Decision Support System TeleRehab in the Management of Post-Stroke Telerehabilitation

**DOI:** 10.3390/life14091059

**Published:** 2024-08-24

**Authors:** Vitaly A. Nikolaev, Alexander A. Nikolaev

**Affiliations:** 1 Research Institute for Healthcare Organization and Medical Management of Moscow Healthcare Department, 9 Sharikopodshipnikovskaya St., Moscow 115088, Russia; 2 Pirogov Russian National Research Medical University, 1 Ostrovityanova St., Moscow 117513, Russia; 3 National University of Science and Technology “MISIS”, 4 Leninsky Prospect, Moscow 119049, Russia; nikolaevopr@mail.ru

**Keywords:** information systems in healthcare, stroke telerehabilitation, decision support system, telemedicine, digital health, post-stroke rehabilitation software

## Abstract

Stroke is the main cause of disability among adults. Decision-making in stroke rehabilitation is increasingly complex; therefore, the use of decision support systems by healthcare providers is becoming a necessity. However, there is a significant lack of software for the management of post-stroke telerehabilitation (TR). This paper presents the results of the developed software “TeleRehab” to support the decision-making of clinicians and healthcare providers in post-stroke TR. We designed a Python-based software with a graphical user interface to manage post-stroke TR. We searched Scopus, ScienceDirect, and PubMed databases to obtain research papers with results of clinical trials for post-stroke TR and to form the knowledge base of the software. The findings show that TeleRehab suggests recommendations for TR to provide practitioners with optimal and real-time support. We observed feasible outcomes of the software based on synthetic data of patients with balance problems, spatial neglect, and upper and lower extremities dysfunctions. Also, the software demonstrated excellent usability and acceptability scores among healthcare professionals.

## 1. Introduction

The COVID-19 pandemic accelerated digital health development and implementation in healthcare [1]. Digital health includes electronic health (eHealth), telehealth, and telemedicine and is generally described as the use of information and communication technologies (ICTs) in healthcare and medicine to improve healthcare service delivery and patient outcomes [2]. Digital health services enhance the quality and efficiency of healthcare, engaging patients in health management [3].

eHealth incorporates all systematic use of ICT in healthcare [4]. The World Health Organization (WHO) defines eHealth as the cost-effective and secure use of ICT in support of health and health-related fields, including healthcare services, health surveillance, health literature, and health education, knowledge, and research [5]. Telehealth is a subcategory of eHealth, meaning any efforts to protect health and prevent an illness using ICT. Telehealth encompasses clinical and non-clinical services, including educational and promotional tools for a healthy lifestyle [4]. Telemedicine mainly focuses on clinical applications, and this is the provision of healthcare services, including remote care and online pharmacies, through the use of ICT in situations where the health professional and the patient are in different locations [4].

Telerehabilitation (TR) is a subfield of telemedicine and can be defined as the remote provision of rehabilitation services via ICT [6]. Globally, according to WHO estimations, about 2.4 billion people are currently living with a health condition that may benefit from rehabilitation, and the estimated need for rehabilitation is only going to increase in the future. In some countries, because of socio-economic reasons and healthcare access problems, more than 50% of people do not receive the rehabilitation services they need [7]. To partially meet this challenge, TR can be used as an additional modality of the healthcare system.

TR is used in different healthcare areas (COVID-19, cardiac cases, knee arthroplasty, chronic diseases, cognitive impairment, stroke, etc.) across the world, demonstrating effectiveness in improving patient outcomes [8,9,10,11,12,13]. Recent studies show that TR can be effectively used to treat stroke, a major cause of disability worldwide [13,14,15,16]. Studies indicate that TR and technology-assisted therapy may have similar or even more pronounced effects than conventional treatment to improve the mobility, balance, speech, and cognition of post-stroke patients [17,18,19,20,21].

TR promotes the accessibility of rehabilitation services to post-stroke patients living in rural areas, reduces travel time, and facilitates early discharge from hospitals. Also, TR improves functional outcomes of patients, lowering long-term disability and associated costs [22]. These observations are in line with another study by Saror, who confirmed that TR could be a successful means for post-stroke interventions [23], improving motor function, balance, and daily life activities. Also, TR can manage the lack of specialized stroke care staff, transportation constraints, and geographic distance [23].

Hao et al., in their review, summarized the virtual-reality-based (VR) TR of 260 participants, indicating its feasibility for post-stroke rehabilitation with a high compliance and adherence rate. Moreover, patients who used VR TR showed high outcomes with respect to upper extremity (Fugl–Meyer Assessment, Box and Block Test), balance functions (Berg Balance Scale), and cognition (Montreal Cognitive Assessment) in clinical and home settings [22].

Cacciante et al. analyzed studies on TR of 132 participants with post-stroke aphasia. The study found that speech and language treatment fits well with TR. They highlighted two technological approaches in the delivery of remote rehabilitation. The “asynchronous” TR does not need the presence of the therapist and hence allows the enhancement of self-administered computer-based exercises, whereas the “synchronous” approach is based on videoconferencing and supports online communication between therapist and patient. Also, the “hybrid” TR combines both approaches (e.g., unsupervised home practice with weekly VC with the therapist) [19].

Another study by Sharififar et al. investigated the impact of TR service on the physical function and adherence of 884 stroke patients [24]. Although TR showed equivalency to conventional treatment in improving functional outcomes across several domains, the authors observed a great diversity of TR methods in type, design, and therapeutic exposure.

Recent reviews on stroke TR have identified a wide variety of tools, technologies, and approaches that are used for the treatment of patients [15,19,24]. They include different hardware systems (e.g., computers, tablets, smartphones, touchscreens, sensors, wearables, cameras, motion tracking devices, computerized boards, etc.) and general and therapeutic software (e.g., video conferencing, exergames, VR, AR, digital avatars, apps, etc.). Moreover, TR is delivered synchronously, asynchronously, or using hybrid formats. Furthermore, healthcare organizations use TR for stroke rehabilitation either as a complementary treatment to conventional therapy or as its alternative [15,19,24].

Stark et al. showed that TR requires a variety of different competencies from healthcare professionals, highlighting the need for an evaluation of existing programs for promoting competencies in the use of TR. Moreover, a competence framework for TR does not yet exist, meaning that the required competencies of healthcare professionals differ depending on the program and performed tasks [25]. This requires an additional tool to assist healthcare professionals in decision-making to manage post-stroke TR.

To improve clinical decision-making, a decision support system (DSS) can be used, which is a computer system that provides decision-making aid during healthcare processes [26]. A clinical DSS in healthcare is a system that provides clinicians and other individuals with knowledge and person-specific information intelligently filtered or presented at appropriate times to improve health and healthcare [27].

Recently, Nugraha et al. show that DSSs have been integrated into various aspects of healthcare (e.g., clinical decision-making, administrative functions), enhancing decision-making, improving the efficiency of clinicians, saving time, reducing manual errors, and optimizing various processes in healthcare departments. Also, they proved that DSSs facilitate complex data-driven decisions and improve accuracy and resource allocation in healthcare. Moreover, healthcare managers highlighted the accessibility of comprehensive data via DSSs to make more informed and precise decisions regarding resource distribution, lowering operational costs, optimizing budgets, and reducing waste [28].

DSSs in healthcare provide clinicians and healthcare managers with relevant evidence-based medical knowledge [29] and support immediate decision-making, bridging a gap between clinical practice and evidence-based medicine. This can improve disease prevention, screening, diagnosis, treatment, and follow-up outcomes [29,30,31,32].

Despite recent growth in research on clinical decision-making systems, the number of studies on post-stroke-related issues is limited [33]. Moreover, there is still a significant lack of software to manage post-stroke TR.

The aim of this paper is to present the results of the development of healthcare software for the management of stroke TR to support decision-making for clinicians and healthcare providers and test its usability. The software can be of practical interest to healthcare professionals and IT experts to implement and promote smart solutions for the sustainable development of healthcare systems. The contribution of this study is the development and testing of the decision support system, “TeleRehab”, that can be used to manage TR after a stroke.

## 2. Materials and Methods

### 2.1. Software Development

We used the Python 3.11 language for coding [34], Tkinter, and Ttk libraries to make a graphical user interface (GUI) of the software. Also, we utilized additional Python-related packages to achieve the functionality of the software. Coding in Python is beneficial as it reduces time and costs for learning and software development. Moreover, Python requires fewer code lines to achieve the same result as compared to other programming languages.

The knowledge base for the DSS was developed using data obtained from international clinical trials on stroke TR, including recent reviews summarizing the most important trends and practices in post-stroke TR [15,35].

First, we used Scopus, ScienceDirect, and PubMed databases to search papers with results of clinical trials for post-stroke TR of patients with upper and lower extremity dysfunctions, balance problems, aphasia, spatial neglect, and other cognitive deteriorations. We used PubMed, as this is an authoritative and free resource to search and retrieve international medical and healthcare literature. The Scopus database was used as an effective search engine for an overview of a topic since ScienceDirect was utilized as the world’s leading source for full texts of scientific, technical, and medical studies.

Second, we analyzed the relevant research and review papers. Then, we extracted data describing healthcare conditions, digital equipment, treatment plans, and recommendations for TR. Finally, we built a knowledge base (KB) to support the decision-making of clinicians and healthcare professionals in managing TR. Also, we embedded information on the KB into the program code.

To provide efficient decision-making for the management of post-stroke patients, healthcare data obtained from typical medical tests and scales is required. Table 1 shows abbreviations of medical tests and scales that we used in the software.

In total, we incorporated 25 stroke-related medical tests and scales into the software.

### 2.2. Simulation Tests

We utilized a randomization method to simulate clinical cases using synthetic data. To test the software, we randomly generated clinical scenarios with respect to age, gender, dysfunction, clinical stroke stage, and medical tests and scale results for virtual patients (Table 2).

The most typical post-stroke dysfunctions (upper/lower extremities, balance problems, aphasia, cognitive deteriorations, upper extremities and cognitive deteriorations, and spatial neglect) were considered in the tests. We obtained synthetic data of 180 patients in the simulation. Then, we entered the data into the software to test the system and obtain healthcare decision-making suggestions.

## 3. Results

### 3.1. The Architecture of the Decision Support System

**Figure 1:**
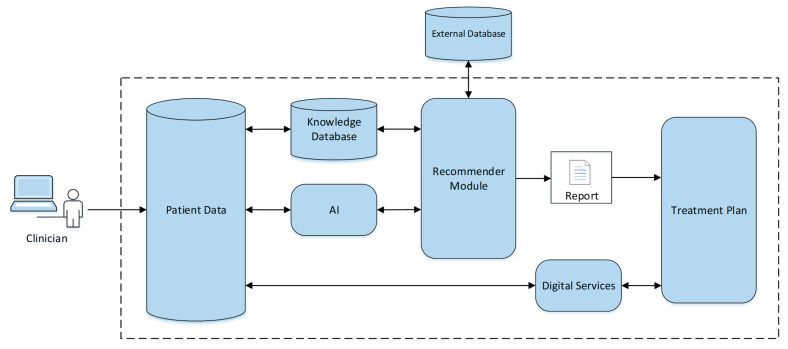
The Architecture of the decision support system.

The application, named TeleRehab, was developed to support the decision-making of healthcare professionals in post-stroke TR. Figure 1 shows the architecture of the DSS.

The DSS has several modules to support decision-making, including patient data, knowledge database, AI, recommender module, digital services, and treatment plan. The DSS generates a report using a recommendation engine that is based on either a knowledge base algorithm or AI. The treatment plan and digital services modules are used to support the functionality of the final step of the electronic healthcare record.

### 3.2. The Software Interface and Functionality

**Figure 2:**
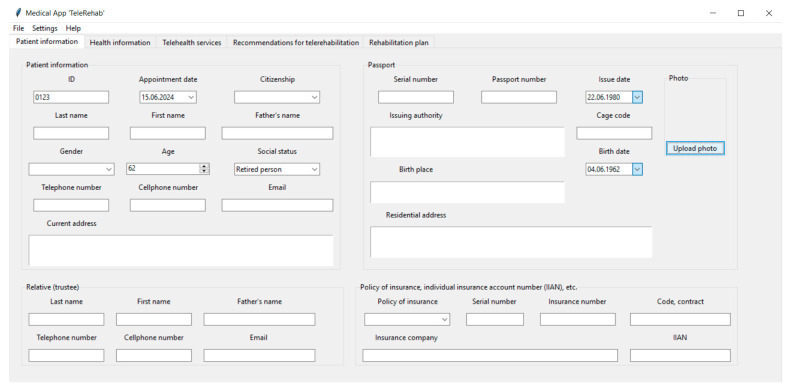
The “Patient information” tab of the GUI.

**Figure 3:**
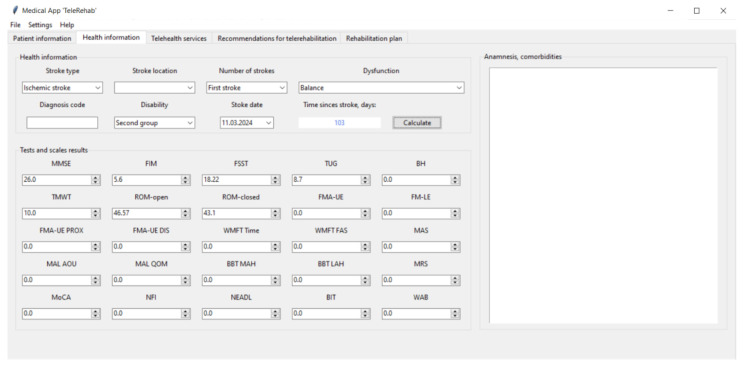
The “Health information” tab of the software.

**Figure 4:**
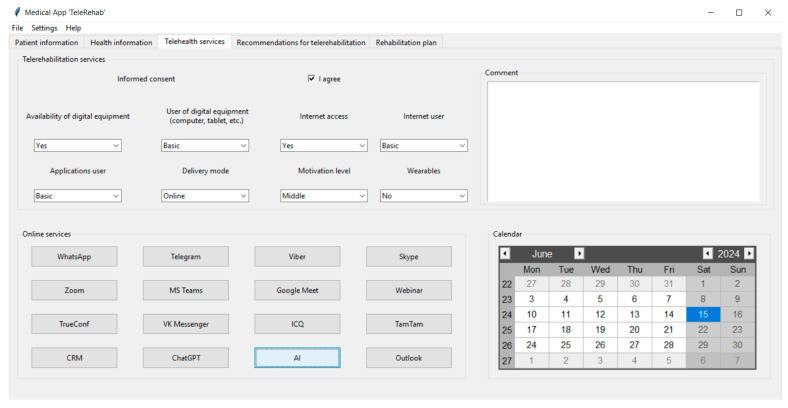
The “Telehealth services” tab of the software.

**Figure 5:**
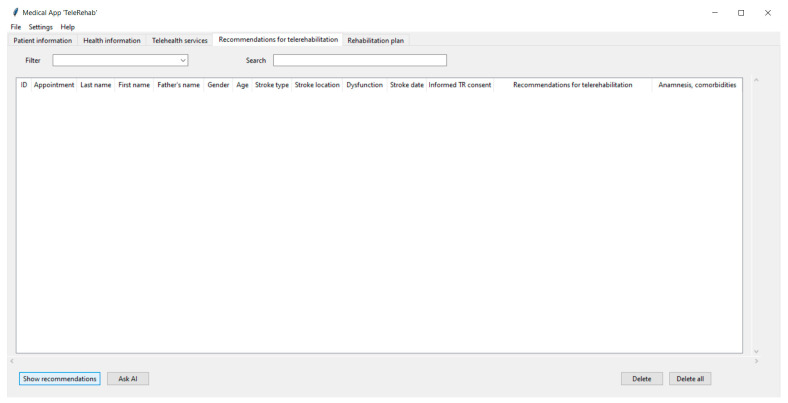
The “Recommendations for telerehabilitation” tab of the software.

Figure 2, Figure 3, Figure 4 and Figure 5 show the graphical user interface (GUI) of the software. The GUI includes registration information, data on symptoms, physical and cognitive examinations, anamnesis, telehealth services, recommendations, and suggestions for TR. The GUI consists of typical IT components (menu and tabs, entry widgets, text widgets, labels, combo boxes, tree view, scrollbars, check buttons, buttons, etc.) to provide a user-friendly interface and functionality.

Figure 2 shows the “Patient information” tab of the GUI.

To use the GUI, a physician enters data or downloads database files. First, the clinician enters the personal data of the patient (ID, citizenship, first name, last name, gender, age, social status, contact information, and appointment date). Then, passport and insurance-related forms are filled out, and a photo of the patient is uploaded. Next, the user enters the contact details of relatives of the patient.

The tab named “Health information” (Figure 3) contains two stroke-related data frames on its left side. Specifically, it aggregates the following data: stroke type (e.g., ischemic, hemorrhagic stroke) and location, number of strokes, dysfunction (e.g., upper extremities, lower extremities, balance, aphasia, spatial neglect, etc.), diagnosis code (e.g., International Statistical Classification of Diseases, local regulations [36]), disability, and stroke date.

To process further, the user clicks the “Calculate” button, and the software calculates the time that has passed since the stroke.

To assign the correct therapy, it is essential to estimate the life quality and rehabilitation potential of post-stroke patients. The “Tests and scales results” frame is designed to accumulate entries with results of post-stroke-specific medical tests (e.g., Fugl–Meyer Assessment, Motor Assessment Scale, Motor Activity Log, Box and Block Test, Montreal Cognitive Assessment, Western Aphasia Battery, Timed Up and Go Test, Mini Mental State Examination, etc.). The DSS utilizes medical test data to provide suggestions for TR. The suggestions for TR contain the description of therapeutic exercises, the treatment regimen (e.g., the dosage, the schedule, and the duration of treatment), the equipment, software, and environmental requirements, the delivery mode, and the source link. Moreover, anamnesis is an important factor in identifying the health problems of post-stroke patients and assigning a therapy. Physicians use the “Anamnesis, comorbidities” widget of the software to record historical healthcare data and comorbidities of the patients.

Figure 4 shows the “Telehealth services” tab that is utilized to provide digital and technical options for TR management. This contains the informed consent of the patient to undergo TR and other telehealth-related information (digital equipment, internet access, digital proficiency level, TR delivery mode, etc.). For instance, the synchronous, asynchronous, or hybrid delivery modes can be used to manage TR and provide personalized treatment recommendations. Also, the software uses settings to indicate to the therapist whether any medical wearables (e.g., trackers, smart watches, heart rate and blood pressure monitors, etc.) are required.

The software allows users to record the level of patients’ motivation for telehealth treatment using the “Motivation level” combo box widget and submit associated comments using the “Comment” textbox widget. The “Online services” frame contains button widgets that are used to run vital online doctor-to-patient and physician-to-physician services, including videoconferencing (VC), messaging, customer relationship management (CRM), and AI services to manage telemedicine. VCs (WhatsApp, Zoom, Skype, etc.) are used to provide telehealth sessions, teleconsultation, and multidisciplinary team meetings. The “ChatGPT” button opens the Chat Generative Pretrained Transformer (ChatGPT, developed by OpenAI) that collects and summarizes data from the internet to provide evidence-based post-stroke rehabilitation program recommendations and advice [37]. The AI button runs another piece of remote AI software to generate clinical recommendations in post-stroke TR. The AI button’s feature set can be customized to perform a variety of AI applications for TR, depending on the healthcare organization’s needs. Additionally, the “Calendar” frame allows the therapist to manage business appointments and time-related prescriptions.

To proceed, the user interacts with the fourth tab, “Recommendations for telerehabilitation” (Figure 5). The main objective of this tab is to generate and show suggestions for TR to physicians and healthcare providers.

The algorithm-based decision is derived based on international clinical trials for stroke TR. They are stored and processed in the knowledge database. To continue, the physician clicks the “Show recommendations” button. Afterward, the window displays a record with the patient’s ID, personal information, short medical data, and suggestions for TR. Additionally, the therapist can open an external knowledge database with suggestions using the “Open file” submenu of the “File” menu. The extra recommendations appear in the same tree view widget. If the software shows several suggestions, the user can search for a specific recommendation using the “Search” and “Filter” fields. The search results that best match the query appear at the top level of the list in the tree view widget. The “Delete” and “Delete all” button widgets are used to remove one, several, or all the items that are shown in the tree view widget.

If the algorithm-based suggestions are not available or outdated, and external databases do not provide relevant information, the clinician can use AI-based recommendations using the “Ask AI” button.

**Figure 6:**
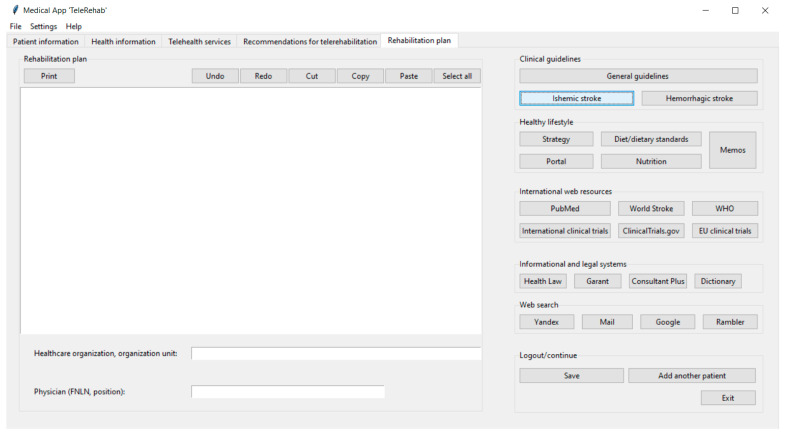
The “Rehabilitation plan” tab of the software.

Finally, the fifth tab, “Rehabilitation plan”, is designed for treatment plan preparation. It contains the “Rehabilitation plan” frame with text and button widgets to type out, edit, and print out a treatment plan (Figure 6). The main text widget is required for writing the treatment plan. To process a hard copy of the treatment plan, the clinician can use the “Print” button to print it out. Additionally, there are five frames containing button widgets to assist therapists in making treatment plans and providing up-to-date recommendations using additional information (clinical guidelines, healthy lifestyles, international web resources, external informational and legal systems, and web search).

Finally, the “Logout/continue” frame contains the button widgets to save entries, continue to work with another patient’s record, or log out. The data can be entered manually or imported from the databases. Also, previously entered data are saved for the next healthcare consultation.

### 3.3. Results of the Simulation

We used synthetic data of 180 patients to test the software. Table 3 summarizes nine cases from a random sample of patients with balance problems, upper/lower extremity disorders, aphasia, cognitive deterioration, and spatial neglect.

The findings show feasible results from simulation tests using the software. The system suggested treatment plans for TR with respect to current clinical trials for all cases. Moreover, the system confirmed the usability of electronic medical record management. The software assists in managing the medical history and personal and healthcare data of post-stroke patients. When a patient requires TR, a medical record can be obtained by a healthcare provider at any time to process the personalized clinical data, prescriptions, and best-suited treatment plan development. The system incorporates ID, name, gender, age, number of strokes, time since stroke, diagnosis, dysfunction, tests (scales) results, and other data to suggest a decision and make a medical record. Furthermore, the software allows a therapist to manage the electronic healthcare records of each patient, including historical data, significantly improving outcomes.

The results also indicate that TeleRehab significantly reduces the time that is required for clinicians to make a decision and to write a treatment plan (*p* < 0.05), increasing the efficiency of healthcare treatment and management (*p* < 0.05).

### 3.4. The Usability Study Results

We recruited 27 healthcare professionals to participate in the study, including 12 males and 15 females. The ages of the participants ranged from 28 to 60 years, with a mean of 43.0 years. We provided the participants with the System Usability Scale (SUS) handouts to test the software. The SUS is a commonly used standardized questionnaire for the assessment of perceived usability, specifically when researchers need a measure of perceived usability [38]. The SUS consists of a questionnaire that provides a reliable tool to assess the usability of the software. Specifically, the SUS became popular because of its reliability with small numbers of participants and because it is rather short [39]. The participants were asked to score the 10 items of the questionnaire with one of five responses that ranged from “Strongly agree” to “Strongly disagree” values [40].

The overall score of the SUS was calculated as follows: First, the score contribution for odd numbers (1, 3, 5, 7, and 9) of the questionnaire was calculated as the scale position minus 1. Second, for even numbers in the questionnaire (2, 4, 6, 8, and 10), the score value was estimated as 5 minus the scale position. Finally, to obtain the overall value of the SUS, we multiplied the sum of the scores by 2.5 [41].

The results of the usability study are shown in Table 4.

The mean overall rating for the SUS was 88.7, which is higher than the average one (68.0). This means that the software demonstrates excellent usability and acceptability among healthcare professionals.

## 4. Discussion

In this study, we showed that TeleRehab software had the capacity to support decision-making for physicians providing post-stroke TR. Also, the developed software was able to generate reliable suggestions for stroke TR, relying on data that were acquired from medical tests and scales of patients and relevant clinical trials on stroke TR. Common input variables included age, gender, stroke duration, stroke type, dysfunction, stroke-related tests and scales, medical comorbidities, and anamnesis.

TeleRehab, as a DSS, is a significant aid to physicians because they face a large number of clinical cases for which a wide variety of digital solutions can be applied. The use of this DSS could assist healthcare providers in making decisions by providing targeted information and offering physicians effective support in TR therapy assignment and management [42].

Consider the basic implications of the developed software. First, our software demonstrates solutions for therapeutic treatment using TR, which is based on the health status of patients and clinical manifestations with respect to specific exercises, their intensity, and the number of repetitions. A distinctive feature of the DSS is that it provides recommendations based on stroke-related test results and patient scores that best fit clinical cases. Also, the system incorporates the availability of digital software and equipment and digital proficiency settings to provide suggestions for TR. For the TeleRehab, we embedded recommendations that were recovered from clinical trials of post-stroke TR. This made it possible for the DSS to provide suggestions to physicians with respect to the clinical cases of the patients. Moreover, each suggestion contained not only a therapy explanation but also a description of the digital equipment, technology, approaches to using them, and source links that are required for healthcare organizations managing TR. As was investigated by Martínez et al., a novel technological implication involves the use of DSSs, aiding therapists in determining the appropriate rehabilitation routine for each patient [43].

Another study reported that the DSS could be integrated into current clinical solutions, with any useful KB for treatment settings potentially driving a standardized way to prescribe the exercises of stroke patients [44]. Also, current clinical post-stroke therapy is aimed at predicting functional improvements at early, middle, and late stroke recovery stages [45].

Second, the DSS allows customization and personalized treatment depending on the availability of hardware and software and the preferences of the patients. The TeleRehab system allows physicians to provide time-bound clinical decisions based on recent advances in clinical knowledge and develop patient-specific treatment plans to customize therapy to the needs and abilities of post-stroke patients. For instance, two patients may have similar diagnoses (clinical cases); one of them owns a personal computer and has access to the Internet, whereas the other has a smartphone only with limited access to the Internet. The software suggests optional solutions to treat both of them. Based on the system recommendations, the physician can assign a synchronous TR (PC, online videoconferencing with a therapist) for the first patient. The other patient will undergo self-administered asynchronous therapy using a mobile app with recommendations for dose and repetition settings. As a result, every patient receives tailored therapy and can perform exercises more comfortably and easily.

Also, as was highlighted by Alasiri et al., our DSS enables healthcare institutions to analyze data from electronic health records and make recommendations to physicians by sending prompts in real time [46]. The designed software allows customization of therapy according to the profile and disability of patients, including integration of the software with electronic health records. Moreover, to support personalized and customized treatment, therapists can use the “Anamnesis, comorbidities” and “Comment” widgets of the software to adjust therapy with respect to medical and family history and environmental factors in semiautomatic or manual mode.

The DSS also supports physicians with clinical guidelines, healthy lifestyle recommendations, and stroke education that can be recommended for patients to improve treatment outcomes and life conditions. Furthermore, the DSS was developed to increase adherence to clinical guidelines on stroke therapy. This is in line with another study [47] claiming that practitioners rarely read, internalize, and implement new guidelines. To improve adherence to clinical guidelines, they suggest a DSS with inbuilt guidelines assisting clinicians with managing patients’ treatment protocols [47]. On a similar note, DSSs educate or remind physicians of the existing guidelines, reducing the need to memorize multiple protocols [48].

Chen et al. observed the potential of DSSs to transform the healthcare system, improve patient outcomes and clinical efficiency, and reduce medical errors and healthcare costs [49]. Other significant implications of the software are as follows: reduced waiting time for appointments, improved medical record management, and reduced inequities in healthcare services for the majority of post-stroke patients.

Typically, therapists must follow up-to-date clinical guidelines to obtain the best therapeutic outcomes. Our DSS incorporates a passive approach requiring users to take actions upon receiving information, suggestions, or advice (e.g., clicking the buttons, opening a tab, etc.) [50]. Also, the software has a proactive approach to a certain degree, resulting in interaction with AI.

In general, our software relates to knowledge-based clinical decision systems. It incorporates rule-based reasoning, capturing knowledge in the form of “if-then-else” statements to achieve suggestions. The DSS uses algorithms for matching patient conditions and healthcare data to the most relevant recommendations. Subject-specific knowledge is recovered from the healthcare literature, including clinical trials and research data from international healthcare organizations [47]. The developed software is in line with the demands of physicians, emphasizing the main benefits of a knowledge-based DSS (e.g., guidance, advice, generation of patient-specific recommendations, etc.) [29].

The information that clinicians need to obtain before making a decision is generated using different “therapist–DSS” interaction scenarios to provide practitioners with optimal and real-time support. Moreover, novel scenarios can lead to the formation of KBs that contain historical data on post-stroke TR, supporting continuous updates.

Hak et al., in their review, emphasized that healthcare information systems are readily absorbing decision support practices to manage clinical knowledge. Moreover, a clinical DSS can be effectively transferred to a digital format, significantly improving the decision-making process [51].

Most clinicians involved in post-stroke rehabilitation are thoroughly acquainted with conventional treatments. However, because of high workloads and rapidly changing technologies, healthcare professionals may not be aware of all trends in the healthcare system. Therefore, the developed software is useful as it eases the work of physicians, lowering the risk of various errors and pitfalls [46]. The automation of the rehabilitation procedure reduces the associated workload of healthcare professionals, allocates more time to other patients, and increases personalization and treatment effectiveness [43].

Moreover, besides the health of the patient, stroke affects all aspects of the patient’s life, meaning the DSS can be used as a tool to suggest lifestyle modifications for post-stroke patients [46,52]. That is why an additional value of our software is to assist healthcare providers in promoting a healthy lifestyle among post-stroke patients. Clinicians need to encourage patients to keep learning about post-stroke lifestyles (e.g., diet, nutrition, habits, exercises, etc.) to improve recovery and provide secondary prevention of mortality. As it was described in the “Results” section, this activity can be performed using the “Rehabilitation plan” tab. To accomplish this purpose, the GUI allows clinicians to use special buttons with built-in links to healthy lifestyle guidelines. Specifically, the button “Strategy” opens the online service with the strategy for promoting a healthy lifestyle among the population. The “Portal” and the “Diet/dietary standards” buttons execute commands to open the healthy lifestyle website and online services with dietary standards. Also, the “Memos” button opens the online service with healthy lifestyle multimedia memos that can be electronically sent to or printed out for patients. This information is useful for healthcare providers to make a comprehensive rehabilitation plan, encouraging patients and their relatives to change their lifestyles to sustain recovery from stroke [46,52].

Moreover, when the information is managed from one place, it allows clinicians and healthcare managers to efficiently analyze data and provide smart and time-efficient decisions on healthy lifestyles and stroke education to patients with stroke.

Another implication of the software accounts for information and law systems aspects as intrinsic components of the information domain of the software. Even though the DSS suggests TR technology for a specific patient according to the clinical case, there might be limitations and restrictions on using TR within a country’s regulatory law. For those reasons, the GUI contains widgets allowing users to work with laws and normative legal documents using online databases. For practical purposes, the software can provide healthcare managers with relevant contract templates for the rental of digital equipment to patients.

Apparently, AI-based solutions for TR and healthcare will demonstrate promising results in decision-making in the near future. That is why another part of our software contains AI solutions. Roosan et al. assessed the effect of ChatGPT in medication therapy management. They observed that AI provided treatment recommendations and general management plans to assist healthcare providers and lower healthcare costs [53]. Haleem et al. highlighted that ChatGPT can be used in healthcare, increasing the efficiency and effectiveness of the healthcare system. Specifically, AI can be utilized in telemedicine services for automation of administrative operations (patient data collection, appointment scheduling, reminders) and for delivering online medical advice [37].

Also, Lee et al., in their research, underlined the importance and promising application of AI in DSSs to support the decision-making of physicians and carry out administration tasks for post-stroke patients. However, the opaqueness of AI algorithms and the lack of user-centric design are considered to be the main challenges for the adoption of AI-based DSSs in healthcare [54].

Moreover, although physicians are increasingly using DSSs in their daily practice, the final decision in healthcare is still made by healthcare professionals, not the information system [30].

This study carries significant implications, underscoring the potential benefits of DSSs in treating post-stroke patients using TR. Also, our research emphasizes the possibility of substantial advantages of such systems to enhance post-stroke rehabilitation and the management of stroke patients.

On a larger scale, according to Epizitone et al., decision-makers and clinicians usually facilitate the need for digitalization in healthcare. Meanwhile, the driving factors at the level of healthcare institutions include administrative, management, and planning functions. The DSS, as a part of the healthcare information system, enables stakeholders (e.g., the government and other players in the healthcare sphere) to grant access to health information, enhancing the delivery of healthcare services [55].

Future studies should be aimed at the development of a DSS for TR after stroke. Other issues to be addressed are managing contradictions between suggestions provided by systems and medical staff, developing seamless integration of the systems into the healthcare sector, and training healthcare providers on using information systems [56,57,58].

## 5. Limitations

Our study has several limitations. First, the automatic mode of the software does not provide suggestions with respect to ethnicity, smoking or other unhealthy habits, family history, or rehabilitation history. However, those suggestions can be found using semiautomatic or manual modes. Next, the KB of the software was built based on clinical solutions found using Scopus, ScienceDirect, and PubMed databases. Also, we did not include other literature sources (books, book chapters, case reports, conference abstracts, and websites) in the search.

Also, we are aware of the usability study limitations because of the small sample size and narrow range of questions.

## 6. Conclusions

We developed a decision support system software, TeleRehab, with its GUI. The Python-based software provides a user-friendly and intuitive interface, allowing for the management of post-stroke TR.

The findings show feasible results from simulation tests using the developed software. The system suggested treatment plans for TR according to healthcare data for all considered cases. Moreover, the system demonstrated its usability for electronic medical records management and practical utility in managing stroke TR to support the decision-making of clinicians and healthcare providers.

These findings suggest that TeleRehab is a prospective DSS that can improve healthcare decision-making in TR and enhance the recovery of post-stroke patients. The software can be easily integrated into the information systems of healthcare organizations and used to manage the treatment of post-stroke patients, significantly reducing the workload of clinicians and costs and improving digital healthcare services.

## Figures and Tables

**Table 1:** Abbreviations of medical tests (scales).

Abbreviation	Test (Scale)
MMSE	Mini Mental State Examination
FIM	Functional Independence Measure
FSST	Four Step Square Test
TUG	Timed Up and Go Test
BH	Broken Hearts Test (Oxford)
TMWT	Ten-Meter Walk Test
ROM-open	Romberg Test Eyes Open
ROM-closed	Romberg Test Eyes Closed
FMA-UE	Fugl–Meyer Assessment for Upper Extremity
FMA-LE	Fugl–Meyer Assessment for Lower Extremity
FMA-UE PROX	Fugl–Meyer Assessment for Upper Extremity Proximity
FMA-UE DIS	Fugl–Meyer Assessment for Upper Extremity Distal
WMFT Time	Wolf Motor Function Test Time
WMFT FAS	Wolf Motor Function Test Functional Ability Score
MAS	Motor Assessment Scale
MAL AOU	Motor Activity Log Amount of Use
MAL QOM	Motor Activity Log Quality of Movement
BBT MAH	Box and Blocks Test for the More Affected
BBT LAH	Box and Blocks Test for the Less Affected Hand
MRS	Modified Rankin Scale
MoCA	Montreal Cognitive Assessment
NFI	Neurobehavioral Functioning Inventory
NEADL	Nottingham Extended Activities of Daily Living Scale
BIT	Behavioral Inattention Test
WAB	Western Aphasia Battery

**Table 2:** Initial conditions for test simulation.

Variable	Value, Description
Age	35–95
Gender	Male, Female
Dysfunction	Upper extremities, Lower extremities, Balance, Aphasia, Cognitive deteriorations, Upper extremities and cognitive deteriorations, Spatial neglect
Clinical stage of stroke	Subacute, Chronic
Medical tests (scales) values	(See Table 1)

**Table 3:** Synthetic healthcare data from a random sample of stroke patients.

Scenario	Age	Gender	Dysfunction	Time Since Stroke, Days	Medical Tests (Scales) Results
Patient 1	62	Male	Balance	103	MMSE 26; FIM 5.6; FSST 18.22; TUG 8.7; TMWT 10; ROM-open 46.57; ROM-closed 43.1
Patient 2	58	Male	Upper extremities	657	MMSE 25; FMA-UE 41; WMFT time 9.17; WMFT FAS 2.86; MAL AOU 1.7; MAL QOM 0.95
Patient 3	62	Female	Upper extremities	475	MMSE 26; FMA-UE 34; WMFT time 11.21; WMFT FAS 2.75; MAL AOU 1.64; MAL QOM 1.32; FMA-UE PROX 25.4; FMAUE dis 12
Patient 4	68	Male	Upper extremities and cognitive deterioration	18	BBT MAH 13; BBT LAH 55; MoCA 20; NFI 187
Patient 5	72	Male	Upper extremities	82	FMA-UE 44; BBT MAH 27
Patient 6	53	Male	Lower extremities	1295	FM-LE 22; FIM 109; MAS 0.8; MRS 3
Patient 7	75	Male	Aphasia	1979	WAB 59.8
Patient 8	71	Female	Cognitive deterioration	3649	MoCA 29; NEADL 16
Patient 9	69	Female	Spatial neglect	3108	BIT 139; BH 45

**Table 4:** The SUS interview questions and results.

No.	Questions	Rating Scale	Mean
1	I think that I would like to use this system	1 (Strongly disagree)–5 (strongly agree)	4.5
2	I found the system unnecessarily complex	1 (Strongly disagree)–5 (strongly agree)	1.4
3	I thought the system was easy to use	1 (Strongly disagree)–5 (strongly agree)	4.2
4	I think that I would need the support of a technical person to be able o use this system	1 (Strongly disagree)–5 (strongly agree)	1.4
5	I found the various functions in the system were well integrated	1 (Strongly disagree)–5 (strongly agree)	4.8
6	I thought there was too much inconsistency in this system	1 (Strongly disagree)–5 (strongly agree)	1.0
7	I would imagine that most people would lean to use this system quickly	1 (Strongly disagree)–5 (strongly agree)	4.4
8	I found the system very cumbersome to use	1 (Strongly disagree)–5 (strongly agree)	1.3
9	I felt very confident using the system	1 (Strongly disagree)–5 (strongly agree)	4.4
10	I needed to learn a lot of things before I could get going with this system	1 (Strongly disagree)–5 (strongly agree)	1.7

## Data Availability

Data available on request from the authors.
